# Barriers to the Use of Clinical Decision Support for the Evaluation of Pulmonary Embolism: Qualitative Interview Study

**DOI:** 10.2196/25046

**Published:** 2021-08-04

**Authors:** Safiya Richardson, Katherine L Dauber-Decker, Thomas McGinn, Douglas P Barnaby, Adithya Cattamanchi, Renee Pekmezaris

**Affiliations:** 1 Donald and Barbara Zucker School of Medicine at Hofstra/Northwell Manhasset, NY United States; 2 Division of Pulmonary and Critical Care Medicine and Partnerships for Research in Implementation Science for Equity (PRISE) Center University of California San Francisco San Francisco, CA United States

**Keywords:** medical informatics, pulmonary embolism, electronic health records, quality improvement, clinical decision support systems

## Abstract

**Background:**

Clinicians often disregard potentially beneficial clinical decision support (CDS).

**Objective:**

In this study, we sought to explore the psychological and behavioral barriers to the use of a CDS tool.

**Methods:**

We conducted a qualitative study involving emergency medicine physicians and physician assistants. A semistructured interview guide was created based on the Capability, Opportunity, and Motivation-Behavior model. Interviews focused on the barriers to the use of a CDS tool built based on Wells’ criteria for pulmonary embolism to assist clinicians in establishing pretest probability of pulmonary embolism before imaging.

**Results:**

Interviews were conducted with 12 clinicians. Six barriers were identified, including (1) Bayesian reasoning, (2) fear of missing a pulmonary embolism, (3) time pressure or cognitive load, (4) gestalt includes Wells’ criteria, (5) missed risk factors, and (6) social pressure.

**Conclusions:**

Clinicians highlighted several important psychological and behavioral barriers to CDS use. Addressing these barriers will be paramount in developing CDS that can meet its potential to transform clinical care.

## Introduction

Clinicians often disregard potentially beneficial clinical decision support (CDS) tools. Extensive study of these tools has shown that their use is associated with a morbidity reduction of 10% to 18%, placing CDS at the top of the spectrum of quality improvement interventions [1]. Improvements in quality of care observed with CDS use [2-8] have been significantly limited by consistently low clinician adoption, estimated at 10% [9,10]. CDS based on Wells’ criteria for pulmonary embolism [11] serves as an illustration of this phenomenon. Systematic reviews have shown that the use of these criteria decreases ordering of computed tomography (CT) scans by 25% without resulting in additional missed pulmonary emboli (PEs) by clinicians [12]. However, clinicians have requested the removal of CDS tools based on these criteria, even when local efficacy has been demonstrated [13].

A systematic review of 58 studies evaluating barriers to clinician adoption of CDS classified these as “CDS specific, organizational, patient and clinician factors” [14]. CDS-specific factors included those that would improve the ease of tool use (ie, minimal mouse clicks, workflow integration). Organizational factors focused on infrastructure and technical issues (ie, having enough computers). Patient factors focused on clinician perceptions of the impact of CDS on the patient-clinician relationship (ie, CDS diminishes the relationship by distracting the clinician). Clinician factors focused on clinician attitudes toward CDS, including a preference for intuitive thought and perception of CDS as a threat to professional autonomy. Clinician attitudes toward CDS, including psychological and behavioral barriers, are not typically addressed during any stage of CDS development although they represent an important barrier to adoption.

Several important publications have detailed the many challenges to CDS reaching its full potential [15], guiding principles for effective CDS [16] and barriers to guideline concordant care and successful implementation of CDS [17-19]. However, improved understanding of the psychological and behavioral barriers to clinician use of potentially transformative CDS tools would assist developers in creating highly adopted, high-impact tools. We sought to explore these barriers by using a comprehensive behavioral framework to interview users of a CDS tool based on Wells’ criteria for pulmonary embolism [20].

## Methods

### Study Design

We conducted a qualitative study involving emergency medicine physicians (residents and attendings) and physician assistants at two large academic health care facilities in New York. The Northwell Health Institutional Review Board approved this study. Informed consent was obtained for all participants. Participants were recruited by email and presentation at regular faculty meetings. Interviews were conducted between June and September of 2019, and each interview lasted from 30 minutes to 1 hour.

### Interview Guide and Behavioral Framework

In-depth interviews focused on the different barriers to use of a CDS tool built based on Wells’ criteria for pulmonary embolism to assist clinicians in establishing pretest probability of PE before imaging. A semistructured interview guide was created based on a comprehensive and parsimonious model of behavior—the Capability, Opportunity, Motivation-Behavior (COM-B) model, which specifies that changing behavior requires changing capability, opportunity, and/or motivation [21]. The COM-B model is at the center of a larger behavioral framework—the Behavior Change Wheel. The Behavior Change Wheel was developed from 19 existing behavioral frameworks and includes 9 intervention functions aimed at addressing deficits in one or more of the conditions described by the COM-B model.

### CDS Tool

The tool was designed to reduce unnecessary computed tomography pulmonary angiography (CTPA) ordering. Additional details about the design, implementation and evaluation of the tool are available in a previous publication [20]. Emergency clinicians entering any electronic order for the diagnosis of PE (D-dimer, ventilation–perfusion [V/Q] scan, or CTPA) are routed to the tool if they answer “yes” to a dialog box asking, “Are you considering PE?” The tool functions as an expanded order set that allows clinicians to formally calculate pretest probability of PE according to Wells’ criteria. For low-risk patients, it only allows clinicians to order D-dimer laboratory testing and for patients with intermediate or high risk of PE, it allows for D-dimer testing, V/Q scan, or CTPA imaging. At any time, the tool can be dismissed by clinicians and then any order can be placed. The tool was developed using adaptive principles in web and health information technology design, which have been detailed in several previous publications [22-25]. The current version of the tool has been active since January 2016 [24]; all study participants had previously used the tool in clinical practice.

### Analysis

Thematic saturation was reached after the twelfth interview, with no new insights obtained by the twelfth participant. The COM-B model informed the development of the interview guide, but it was not used to create a priori themes before qualitative analysis. Inductive methods were used to analyze session notes and audio recordings with the COM-B model as a guiding theory. We identified themes using open and then axial coding, and we coded our data accordingly using the qualitative data analysis software NVivo (version 12, released 2018; QSR International Pty Ltd.). Two members (SR and KLD) of the study team, with experience conducting qualitative analysis, coded all sessions. All discrepancies were resolved by consensus.

## Results

Interviews were conducted with 5 resident physicians, 5 attending physicians, and 2 physician assistants. Six major barriers to tool use were identified, including (1) Bayesian reasoning, (2) fear of missing a PE, (3) time pressure or cognitive load, (4) gestalt includes Wells’ criteria, (5) missed risk factors, and (6) social pressure (Table 1).

**Table 1:** Themes and representative quotes from qualitative interviews with clinicians.

Theme	Quotes
Bayesian reasoning	“I don’t think [pre-test probability] matters for the CT scan…I’ve been told if you order a CT, you’ll either see it or you won’t.”
Fear of missing pulmonary embolism	“…the environment with [quality improvement oversite] and the medical-legal situation, I might argue the threshold to test here is 0%.” “A lot of people say that I'd rather order 10 extra CTs than miss 1 PE…There is a culture of fear of missing.” “…as I’ve been in practice and I’ve had law suits and I’ve seen people have lawsuits…I feel like I tend to irradiate more people than I would have like as a resident…And now I’m like ok radiation, its good stuff.”
Time pressure or cognitive load	“I think that the biggest takeaway that you could take from interviewing ER providers is time, like that’s the thing that matters most to us. Time and like ease of use.” “[PERC] feels good…and it’s shorter…Wells’…it’s longer, it takes a little bit more mental energy to go through.”
Gestalt includes Wells’ criteria	“I never use [the clinical decision support tool], I have done the scoring in my head.”
Missed risk factors	“[M]y clinical gestalt has red flags for things that are not on Wells’. …it doesn’t have some of the younger woman risk factors like OCPs [oral contraceptive pills] and smoking history.”
Social pressure	“[I]t does happen once in a while that I’ll think this person, the patient, can get away with a D-dimer alone but the [physician assistant] or the learner wants to do a CT Scan, and I’m not averse to letting that go through because… sometimes you just need to get talked out of it by getting enough negative ones.” “…I think patient expectations are different. Emergency medicine is becoming like…it’s all about customer service. …A lot of things you do because you know your patients are…expecting it.”

Clinicians highlighted the belief that the tool was not useful to them because all elements of Wells’ criteria for pulmonary embolism were incorporated into their gestalt. The clinical prediction rule is well known and commonly taught during training in emergency medicine. Fear of missing PE was another major theme identified in our analysis. Patient health consequences were rarely mentioned. Clinicians felt that missed PEs were likely to be less clinically significant and unlikely to result in significant harm to patients, but they worried they still might trigger department quality improvement review or legal action. Time pressure was also highlighted as a major barrier to tool use. Although clinicians denied that cognitive load kept them from using the tool, the majority of clinicians spontaneously mentioned their preference for the pulmonary embolism rule-out criteria (PERC) owing to its simplicity. PERC is validated for use in low-risk patients to rule out PE if eight criteria are negative [26].

Additional themes included Bayesian reasoning, missed risk factors, and social pressure. Bayesian reasoning reflected some clinicians not recalling that the posttest probability of PE would be impacted by the pretest probability of PE, predicted by the CDS, regardless of the results of the CT scan. Missed risk factors reflected clinicians’ mistrust of the CDS as Wells’ criteria for pulmonary embolism do not explicitly include a few known risk factors for PE. Social pressure reflected many clinicians’ report that other members of the care team, including the patient and their primary care doctor, could influence their decision to not use or not follow the recommendation of the tool.

## Discussion

### Principal Findings

In this qualitative study of barriers to the use of CDS for the evaluation of PE, participants reported that the CDS tool was not useful to them despite decades of research validating the efficacy of the clinical prediction rule that served as the basis for the tool and our work showing that tool users at our institution improved their CT scan ordering behaviors [20]. Most clinicians felt that they were able to incorporate the elements of the Wells’ criteria for pulmonary embolism into their decision-making without using the tool. The clinical prediction rule, with seven elements, each weighted differently, is complicated enough to make memorization unreliable. There is evidence that clinicians have trouble remembering even simple clinical prediction rules. For example, a study in which clinicians were surveyed about their knowledge of the Ottawa Ankle Rule found that although 89.6% reported using the rule always or most of the time in appropriate circumstances, only 30.9% correctly remembered which four components were part of the rule [27].

Another major barrier to tool use was fear of missing a PE. In a previous study, surveyed emergency medicine clinicians said that about one-fifth of all imaging studies ordered were medically unnecessary [28]. The main perceived contributors were fear of missing a low-probability diagnosis and fear of litigation. Interestingly, although many clinicians in our study reported this as a barrier, only one knew of any emergency medicine clinician who had ever been sued for a missed PE. The great majority of patients in New York who sustain a medical injury because of negligence do not sue [29,30], and evidence of adherence to known clinical practice guidelines can help clinicians avoid liability [31]. More importantly, systematic reviews have shown that the use of the Wells’ criteria for pulmonary embolism decreases CT scan ordering by 25% without resulting in additional missed PEs by clinicians [12]. These facts were not unknown to clinicians in our study, and many volunteered similar statements. However, these facts alone were not enough to address this important psychological barrier to tool use.

Psychological and behavioral barriers, such as gestalt includes Wells’ criteria and fear of missing PE, as well as time pressure or cognitive load are not easily addressed by educational quality improvement interventions. Emergency medicine clinicians are familiar with and believe the Wells’ criteria for pulmonary embolism are useful, as evidenced by a study which surveyed clinicians at our institution [32]. Additionally, the benefits of using the CDS tool, which incorporates these criteria, were reviewed in several academic detailing training sessions for the tool with clinicians before its launch [20]. Additional educational sessions would not be likely to address the sense for physicians that their gestalt adequately considers Wells’ criteria for pulmonary embolism without referencing them. This is likely to be the case as well for using educational sessions or traditional CDS to reduce fear of missing PE.

Time pressure or cognitive load may be the most difficult to address and an important barrier to the use of CDS in the emergency department. However, clinicians reported that low utility was the driving factor for dismissal, and not cognitive load or time. They also reported the importance of eliminating even a single extra click and a strong preference for PERC owing to its simplicity; however, unlike the Wells’ criteria for pulmonary embolism*,* it can only be used in low-risk patients. Additionally, emergency medicine may be the clinical specialty with the highest task load and one of the highest cognitive loads [33]. This demanding environment exerts strong pressure on clinicians to find the fastest, safest path forward. In the case of assessment for PE, this often means skipping the CDS and ordering a CTPA—the definitive test to evaluate for PE.

Some of the barriers identified by this study, such as Bayesian reasoning and missed risk factors, might be addressed by simple educational quality improvement interventions. Addressing common knowledge gaps with education—that is, the role of Bayesian reasoning and instances when the rule is not valid—may help to increase adoption rates. A recent study of guideline-discordant CT scans performed to evaluate for PE found that in 39% of these cases, patients had risk factors that were not explicitly incorporated in traditional clinical prediction rules [34]. Building tools with brief instruction manuals may help clarify for clinicians when to use and when not to use these tools. Additionally, although educational quality improvement interventions would be less likely to address barriers such as gestalt includes Wells’ criteria, fear of missing PE, and time pressure or cognitive load, there are several behavioral interventions that might move the needle. For example, tool endorsement by key leadership might increase use, by communicating institutional backing for tool use and mitigating the fear of missing PE. Avenues to address the social pressure barrier would need to be informed by further research, for example, by knowledge of study patients and their preferences.

We have shown how a behavioral model can identify novel barriers to the adoption of a CDS tool. Our findings underscore the importance of addressing the psychological and behavioral barriers to CDS use. Although the field stands to benefit greatly from much anticipated advances in computational capabilities—for example, artificial intelligence, including machine learning—these tools are unlikely to meet their potential to transform clinical care until behavioral barriers to their use are adequately described and addressed.

### Limitations

Our work has several limitations. All clinicians work in the New York City metropolitan area. Both institutions are academic tertiary care centers. Clinicians outside of this geographical area or working in community hospital settings were not included in this study.

### Conclusions

In summary, clinicians highlighted several important psychological and behavioral barriers to CDS use. Addressing these barriers will be paramount in developing CDS that can meet its potential to transform clinical care.

## Abbreviations

CDS: clinical decision support
COM-B: Capability, Opportunity, and Motivation-Behavior
CT: computed tomography
CTPA: computed tomography pulmonary angiography
PE: pulmonary embolism
PERC: pulmonary embolism rule-out criteria
V/Q: ventilation–perfusion
